# Analysis of the composition, characteristics, and antifungal properties of cutin in goji berry fruits at different developmental stages

**DOI:** 10.3389/fpls.2025.1528881

**Published:** 2025-02-11

**Authors:** Yueli Zhou, Dayuan Chen, Cong Wang, Huaiyu Zhang, Lunaike Zhao, Junjie Wang, Qiding Peng

**Affiliations:** Key Laboratory of Storage and Processing of Plant Agro-Products, School of Biological Science and Engineering, North Minzu University, Yinchuan, China

**Keywords:** *Lycium barbarum* L., cutin, chemical composition, *Alternaria alternata*, antifungal activity

## Abstract

Cutin is the main component of the fruit cuticle framework and plays a role in resisting biological stress. However, little is known about the cutin composition and antifungal properties of goji berry (*Lycium barbarum* L.). In the current study, paraffin sections and gas chromatography/mass spectrometry (GC/MS) techniques were used to identify differences in cuticle structure and chemical composition of Ningqi-1 and Ningqi-5 goji berries at different developmental stages. Meanwhile, cutin extracts from goji berries at four developmental stages were evaluated for their effects on spore germination, germ tube elongation, and mycelial growth of *A. alternata*. Twenty-six cutin compounds were identified in Ningqi-1 and Ningqi-5. Fatty acids, alkanes, aromatic acids, and small molecule acids were the main components of goji berry cutin, which are related to the formation of cutin structures. Spore germination and germ tube elongation in *A. alternata* were significantly inhibited by treatment with cutin extracts from goji berries at different developmental stages. Moreover, the cutin monomer content in goji berries may be closely related to antifungal properties. This study provides a research basis for further investigation of the accumulation mechanism of natural antifungal substances during the growth and development of goji berries.

## 1 Introduction

Plant cuticle ubiquitously covers the outer layer of the cell wall, with cutin serving as its primary structural component. Cutin is a complex mixture, primarily composed of molecules with chain lengths of 16 or 18 carbons and their derivatives, mainly including fatty acids, diacids, and aromatic compounds. The cutin of pitaya fruits (*Hylocereus polyrhizus*) is mainly composed of 9(10),16-2-OH-cetanoic acid and 9,10-epoxy-ω-OH-occidanoic acid (Huang and Jiang, 2019). In the leaves and fruits of tomatoes, the main hydroxy fatty acid is 10,16-dihydroxycetanoic acid (Fich et al., 2016). Therefore, the chemical composition and content of cutin vary across plant species and tissues (Järvinen et al., 2010).

Cutin plays a critical role in defining organ boundaries and is an essential component of plant responses to abiotic and biotic stresses, which can mitigate water loss and UV damage and protect plants from microbial invasion (Kurdyukov et al., 2006; Rozema et al., 2009; Tafolla-Arellano et al., 2018; Fawke et al., 2019; Xin and Fry, 2021). It had been reported that the cutin content of sweet cherries reportedly did not change 40–85 days after flowering despite the surface area of the fruit increasing twofold, indicating that the existing cutin skeleton could be rearranged to adapt to expansion without cracking (Peschel et al., 2007; Segado et al., 2020). The locust bean gum (Lbg) coating of cutin monomers extracted from tomato-processing byproducts can effectively control cherry tomato fungal decay (Aloui et al., 2021). Despite this application, there are few reports on cuticle modifications that occur during the life span of goji berry (*L. barbarum* L.), and reports have often focused on cuticular wax rather than cutin.

Goji berry is a fruit with high nutritional and medicinal value, widely cultivated in northwest China. However, postharvest goji berries are susceptible to fungal rotting, such as infection by *Alternaria alternata*, due to their thin skin, high juice content, and abundant nutrients (Yuan et al., 2012; Zhang et al., 2021; Vidović et al., 2022). Ningqi-1 is a classic variety of goji berry, while Ningqi-5, a fresh food variety, is considered an improvement over the former in terms of quality and adaptability (Ma et al., 2009; Qin et al., 2012). Our previous study demonstrated that the sensitivity of two goji berry varieties to *A. alternata* differed, with Ningqi-5 being more sensitive to *A. alternata* than Ningqi-1 (Wang et al., 2021). Additionally, it was observed that the cuticular wax of goji berries exhibits strong resistance to black rot (Wang et al., 2021). However, it remains unclear whether the presence of cutin influences this resistance.

*A. alternata* is a pathogenic fungus that can infect fruits such as goji berries (Wang et al., 2021), pistachios (Ozkilinc and Sevinc, 2018), lemons (Jurick et al., 2014), and various citrus varieties (Zhu and Xiao, 2015). The infection of these fruits not only causes significant economic losses but also poses health risks due to the production of various toxins. Studies have reported that pre-harvest infection involving latent pathogenic may be associated with the structural composition of the cuticular layers (Reisige et al., 2006; Altieri et al., 2007; Ding et al., 2018). The presence of C28 aldehyde in the epidermal wax of wheat leaves has been shown to induce structural differentiation in *Puccinia graminis* f. sp. *tritici* during infection, and cutin monomers can be involved in triggering adsorption germ tube elongation (Reisige et al., 2006). In addition, hexadecanoic acid, a component of cutin, exhibited a stronger inhibitory effect on *Aspergillus* than on *Penicillium* (Altieri et al., 2007; Ding et al., 2018). However, there are no reports indicating that the growth and development of *A. alternata* are affected by the cutin of goji berries.

Bionics is a multidisciplinary field that studies and replicates designs found in nature, often applying these principles to food packaging and other areas. The leaf-inspired bionic antifungal adhesion film incorporating beeswax has significant physicochemical properties, and its antifungal and antibacterial adhesion capabilities extend the shelf life of fruits and vegetables (Song et al., 2020; Lei et al., 2021; Huang et al., 2022). Chitosan-based biofilms have been successfully used as packaging materials for various food products, promoting preservation and prolonged quality (Mohammed, 2010). Cutin is the primary contact area between plants and the environment. Its complex composition is a potential source of inspiration in the field of bionics, and it is widely used in the preservation of fruits and vegetables. For example, many metal food packaging interiors are coated with materials derived from tomato cuticles (Gandini et al., 2016; Heredia-Guerrero et al., 2017), and the application of Lbg/cuticle monomer can reduce the decay rate of tomatoes by 55%–60% (Aloui et al., 2021). However, the chemical composition of goji berry cutin remains unclear, and whether it can be developed into a bionic film for food preservation remains to be determined.

In the current study, paraffin sections and gas chromatography/mass spectrometry (GC/MS) techniques were used to identify differences in cuticle structure and chemical composition of Ningqi-1 and Ningqi-5 goji berries at different developmental stages. Meanwhile, cutin extracts from goji berries at four developmental stages were evaluated for their effects on spore germination, germ tube elongation, and mycelial growth of *A. alternata*. These experiments aimed to provide deeper insight into antifungal mechanisms and explore the potential application of goji berry-derived materials to promote freshness and extend shelf life.

## 2 Materials and methods

### 2.1 Plant and fungal material

Two varieties of cultivated goji berries (*L. barbarum* L. cv. Ningqi-1 and Ningqi-5) were hand-harvested from the Ningxia Academy of Agricultural and Forestry Sciences in Yinchuan, China (106.18°E, 38.65°N). The selected goji berries were uniform in shape and size, with no visible physical damage or microbial infection. The fungal pathogen *A. alternata* was isolated from infected goji berries and cultured on potato dextrose agar (PDA) at 28°C for 1 week (Zhao et al., 2023).

### 2.2 Treatment and method

#### 2.2.1 Sample label acquisition and collection

The method described by Zhao et al. (2021) was used to mark fruits at different developmental stages. All goji berry trees used for fruit harvesting were over 6 years old. The goji berry development and maturation were categorized into four stages: young fruit, green fruit, turning fruit, and red fruit, which corresponded to the 7th, 14th, 28th, and 35th days after flowering, respectively. Marking lines were tied at the junction of the fruit and branch during the peak flowering period, and different colored marking lines were used to distinguish them every other day. These color-coded marking lines were used to track the growth and development stages of the goji berries during sampling. Finally, fruits free of pests and diseases were randomly selected.

#### 2.2.2 Separation and identification of the components of fruit cutin

Calculation of the fruit surface area was performed in accordance with the scanning method described in Wang et al. (2014; 2021). The extraction of fruit cutin was performed following the method described by Wang et al. (2016), with minor modifications. Whole goji berry fruits were boiled in water and quickly transferred to ice water; then, the fruit cuticle was peeled off using tweezers. The cuticle samples were immersed in an oxalic acid buffer (4 g/L) containing ammonium oxalate (16 g/L) and placed in a shaking incubator at 120 rpm and 48°C for 1 day. Afterward, the samples were thoroughly rinsed with deionized water, dried, and ground. Enzymatic hydrolysis was conducted at 35°C for 12 h using a 20 mM citric acid buffer (pH 3.7) containing 0.1% cellulase and 0.5% pectinase. The samples were then rinsed with deionized water and dried, followed by treatment with isopropyl alcohol at 85°C for 10 min. Overnight dewaxing was then conducted using isopropyl alcohol, chloroform/methanol (2:1), chloroform/methanol (1:2), and methanol. The dewaxed samples were then dried under mild nitrogen and stored in a desiccator. Cutin extraction was repeated three times for each cultivar sample.

#### 2.2.3 Sample pretreatment

Before GC/MS analysis, a 10-mg sample of the cuticle layer was transesterized in a 14% boron trifluoride and methanol solution at 70°C for 16 h. The mixture was cooled and filtered, and three times the volume of saturated sodium bicarbonate aqueous solution was added to terminate the reaction. A 200-μL *n*-tetracosane (1 μg/μL) was used as an internal standard. The resulting mixture was extracted with chloroform, and the combined organic phase was dried with Na_2_SO_4_ and blown dry in a mild nitrogen stream at 40°C. Then, 100 μL of pyridine and 100 μL of bis(trimethylsilyl)trifluoroacetamide (BSTFA) were added, and the preparation was incubated at 80°C for 60 min. The sample was then dried under a gentle stream of nitrogen and dissolved in 1 mL of chloroform. Before GC/MS analysis, the solution was filtered through a 0.22-μm microporous membrane. The prepared samples were stored at −20°C prior to analysis.

#### 2.2.4 GC/MS analysis

GC/MS analysis was performed using a gas chromatograph (Agilent, Santa Clara, CA, USA) and a mass spectrometer (Agilent). Compound separation was achieved using a DB-1 MS capillary column (30 m × 0.25 mm, 0.25 μm). The helium gas flow rate was 1.0 mL/min. The GC parameters had injection temperature of 280°C, mass spectrometry source temperature of 230°C, column flow rate of 1.0 mL/min, aux-2 temperature of 280°C, splitless injection, and injection volume of 1 μL. The MS parameters were electron bombardment ionization mode, ionization voltage of 70 eV, ion source temperature of 270°C, MS source temperature of 250°C, quadrupole temperature of 150°C, full scan mode *m*/*z* range 50–650, and solvent delay of 5 min. The heating program was set to 70°C for 1 min, increased to 200°C at a rate of 10°C/min, held for 2 min, increased to 290°C at a rate of 3°C/min, then increased to 300°C at a rate of 2°C/min, and held for 5 min. Cutin compounds were detected via the NIST 2014 library or by comparing their mass spectra and relative retention index with standards. Saturated alkanes were detected using the external standard method, and other compounds were identified via comparisons with known amounts of the internal standard *n*-tetracosane.


(1) $$\text{Cutin content }\left(\text{μg}/{\text{cm}}^{2}\right)=\left[(0.2\text{ μg}/\text{μL}{)}^{*}{\text{S}}_{2}/{\text{S}}_{1}{\;}^{*}1^{*}\;1,000^{*}\text{M}/\left(10\text{ mg}\right)\right]/\left({\text{N}}^{*}\text{S}\right)$$

Corrected: Calculation of the single of a content substance

Goji berry fruit surface area: S (cm^2^)

Standard peak area: S_1_

Individual mass peak area: S_2_

Standard concentration: C standard = 1 μg/μL

Standard volume: V = 200 μL

Number of goji berries: N

The injection volume was 1 μL, and the total volume of the sample was 1,000 μL.

Goji berry cutin percentage calculation:

The proportion of a substance = the percentage of the substance × (1 + percentage of the standard substance). According to the peak area of the compound, the relative content of the compound was determined via the area normalization method.

#### 2.2.5 Staining paraffin sections

Paraffin sections were generated as described in Wang et al. (2016) and Wu et al. (2022), with minor modifications. Samples were fixed in paraformaldehyde solution and then dehydrated in a graded ethanol series. The dehydration process included treatment with 100% ethanol for 30 min, followed by immersion in a mixture of anhydrous ethanol and xylene (1:1) for 20 min, and then two rounds of clearing in pure xylene for 10 min. Filtration after dissolution. In the next procedure, xylene and soft wax (1:1) were immersed at 58°C for 40 min; soft wax and geocerite were immersed at 58°C for 1 h, embedded, and sliced. They were stretched in a water bath for 3 min and removed from the water. After Safranin O/Fast Green staining, each section was sealed with glycerin gelatin and observed.

#### 2.2.6 Effects of cutin extract and monomer on *A. alternata*

Different concentrations of cutin monomer solution (400 µL) were added to water agar slides and evenly spread. A chloroform solution was used as the control. A 10-µL aliquot of 1 × 10^6^/mL of *A. alternata* spore suspension was added to the surface of the slide. The numbers of germinated spores and germ tube lengths were recorded at 2 h, 4 h, 6 h, and 8 h. In addition, *A. alternata* cakes were placed in the center of the PDA medium (20 mL) and then incubated at 28°C and 85%–95% relative humidity (RH). Colony diameters were measured, and antifungal rates were calculated of *A. alternata* every 2 days. Each treatment was repeated three times.

#### 2.2.7 Statistical analysis

All experiments were conducted three times, and results were analyzed using GraphPad Prism 5.0 software and one-way analysis of variance (SPSS 25.0). Mean differences were compared using Duncan’s multiple range tests, with a significance level of *p* < 0.05.

## 3 Results

### 3.1 Microscopic investigation of goji fruit cutin

**Figure 1:**
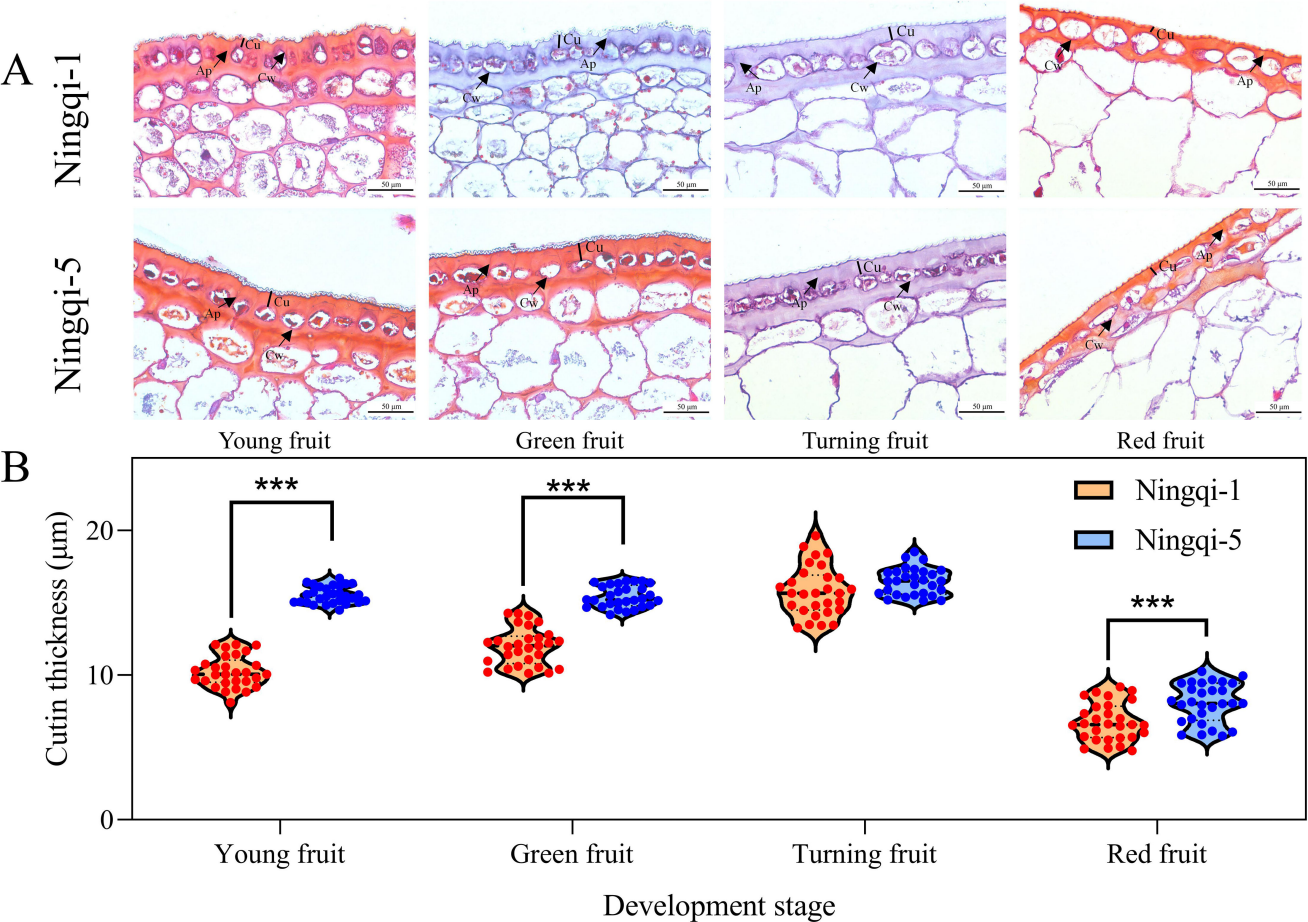
**(A)** Epidermal microstructure of goji berry fruits during development. **(B)** Cutin thickness. Cu, cutin; Ap, anticlinal wall cuticular peg; Cw, cell wall. “***” p < 0.001.

Microscopic observation results indicated that the cutin thickness in both Ningqi-1 and Ningqi-5 initially increased and then decreased as the fruit developed and matured (**Figure 1**). In both cultivars, the cuticle structures were dense, and the outer epidermal cells were small in diameter, oval, and closely arranged. From the plant epidermis to the tissue, the cell density gradually decreased, the shapes changed from oval to irregular thin-walled cells, and cellular content increased. A comparison between the two cultivars indicated that the cell density in Ningqi-5 was lower than that in Ningqi-1. In addition, the cutin thicknesses of Ningqi-1 fruits in the young (10.23 ± 1.07 μm), green (105.82 ± 1.69 μm), and red (6.76 ± 1.29 μm) stages were significantly thinner than those of Ningqi-5.

### 3.2 Analysis of monomer components in goji berry cutin extracts

#### 3.2.1 Analysis of total chemical constituents in goji berry cutin extracts

**Figure 2:**
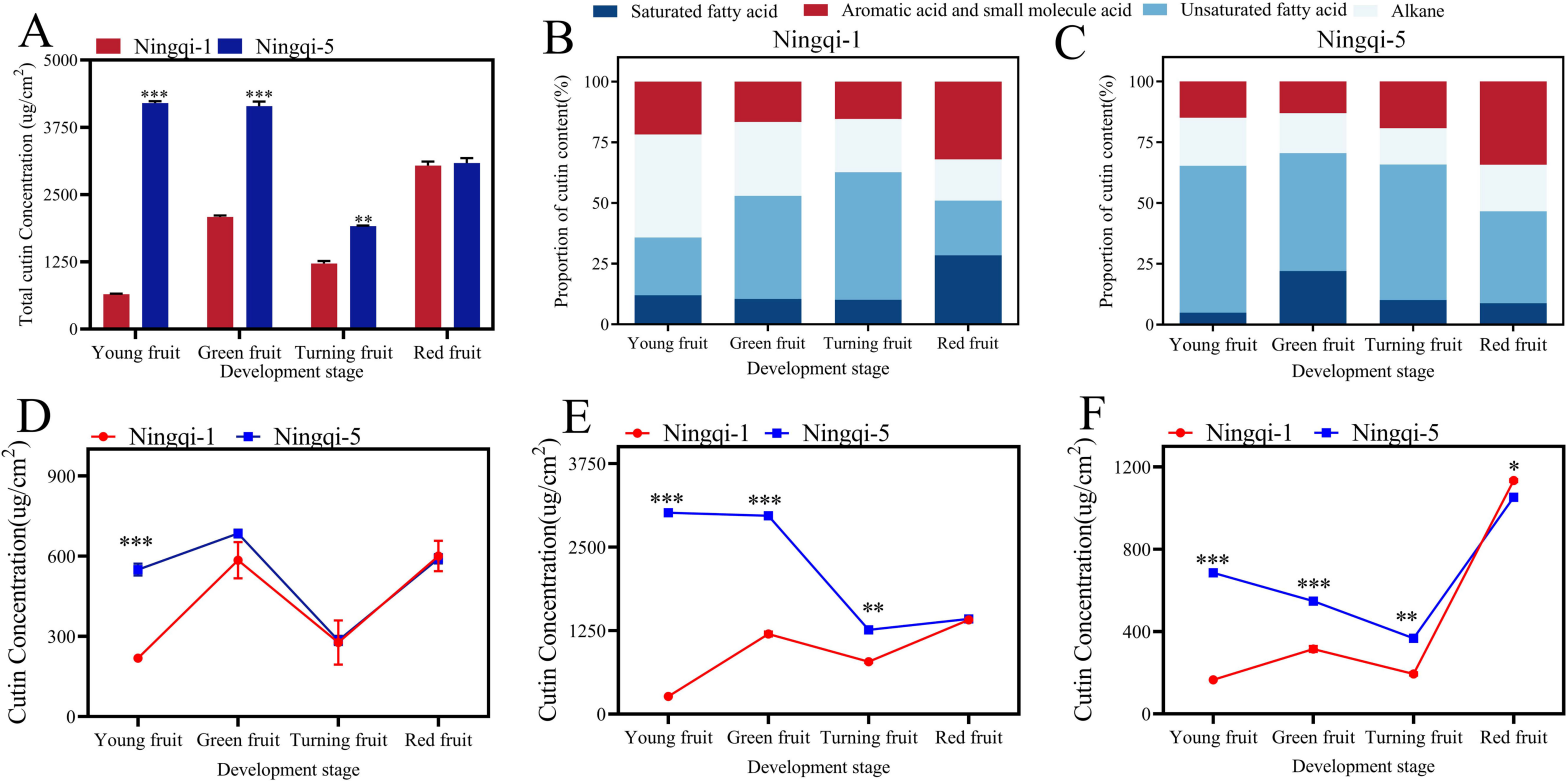
Cutin composition and content profiles in goji berries during the four spatial growth stages. **(A)** Total cutin content. **(B)** Proportion of each component and content change of each component in Ningqi-1 fruits. **(C)** Proportion of each component and content change of each component in Ningqi-5 fruits. **(D)** Alkanes. **(E)** Fatty acids. **(F)** Aromatic acids and small molecule acids. “*” *p* < 0.05, “**” *p* < 0.01, “***” *p* < 0.001. Error bars indicate the mean ± the standard error of the mean.

The analysis revealed that goji berry cutin was mainly composed of C16 and C18 hydroxy fatty acids. The cutin yields of fruit at different developmental stages are shown in **Figure 2A** and **Supplementary Table S1**. Total cutin content was highest in the young, green, and turning stages of Ningqi-5 fruits, whereas red fruits had a similar cutin content across both cultivars (**Figure 2A**). In young fruits, Ningqi-5 had the highest cutin content, while Ningqi-1 had the lowest cutin content (4,202.17 ± 35.51 μg/cm^2^ vs. 646.86 ± 11.96 μg/cm^2^). As the fruit matured, the cutin content of Ningqi-5 decreased to 1,913.54 ± 10.49 μg/cm^2^, whereas that of Ningqi-1 increased to 3,041.18 ± 73.42 μg/cm^2^ (**Figure 2A**). Distinct differences in the proportions of cutin compounds were observed between the two cultivars. Fatty acid was the main cutin component in the two goji berry cultivars. Unsaturated fatty acids were the predominant abundant compounds in Ningqi-1 fruits, accounting for 52.56% of the total cutin content. With fruit maturation, the proportion of saturated fatty acids increased from 10.11% to 28.46%, while the proportion of alkane increased from 16.94% to 42.48% (**Figure 2B**). Similar trends were observed in Ningqi-5: as the fruit matured, the total cutin content decreased, while the proportion of unsaturated fatty acids increased from 37.80% to 60.38% (**Figure 2C**).

#### 3.2.2 Changes in alkane content

**Figure 3:**
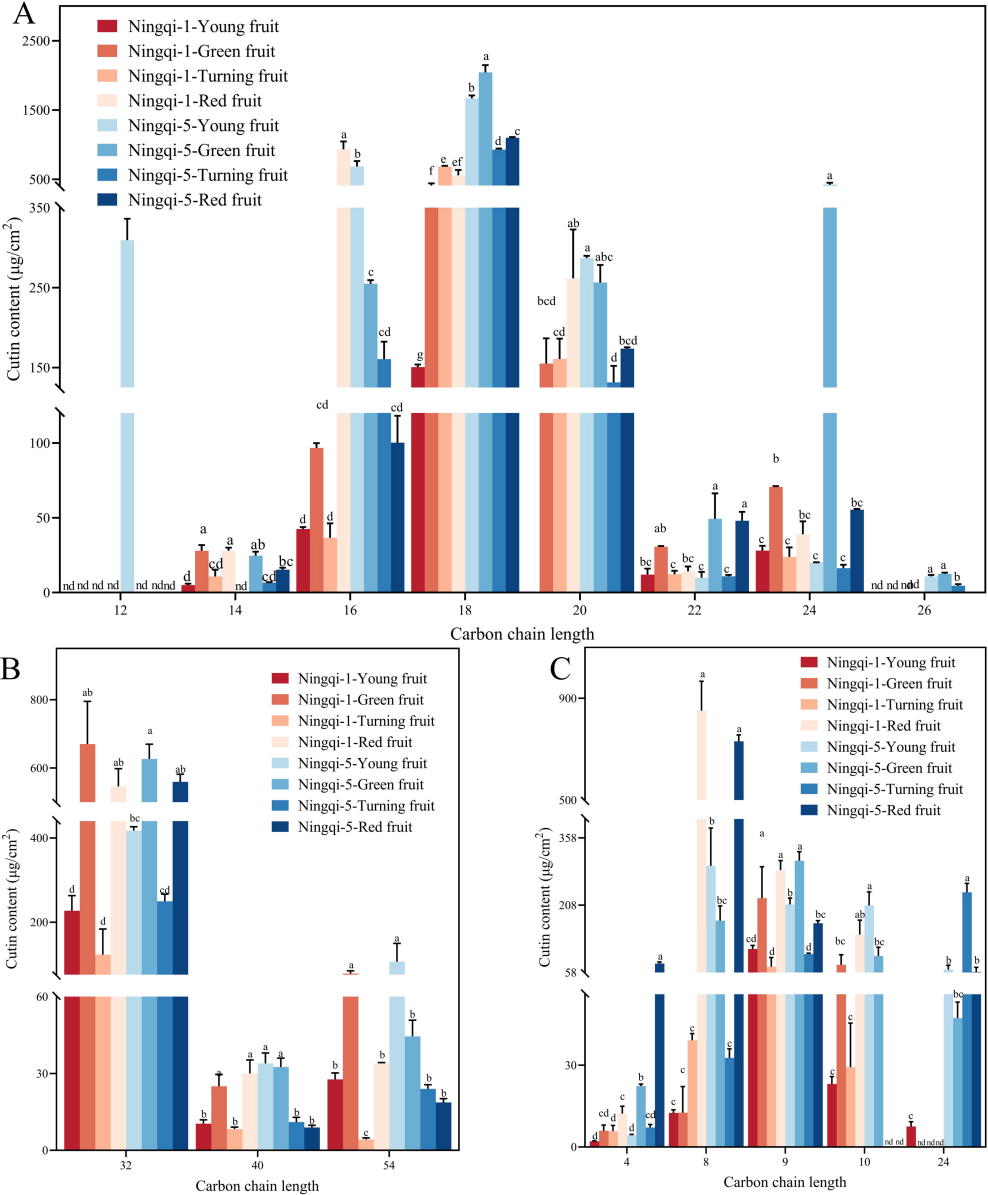
The lengths of fatty acid carbon chains **(A)**, alkanes **(B)**, and aromatic acid and small molecule acid cutin monomers **(C)** changed during development. Different letters during the same developmental stage differ significantly (*p* < 0.05).

Three compounds were detected in goji berry fruits at the four different stages of development: *n*-dotriacontane, *n*-tetracontane, and *n*-tetrapentacontane (**Figure 2D**; **Supplementary Table S1**). Alkane content was the least abundant compound in Ningqi-1 young fruits, accounting for 284.77 ± 16.73 μg/cm^2^. However, as Ningqi-1 fruit maturity increased, alkane content increased to 600.24 ± 56.82 μg/cm^2^. A similar upward trend was observed in Ningqi-5 fruits, where alkane content rose from 284.77 to 684.79 μg/cm^2^. Significant differences in alkane content were evident at the young fruit stage between the two cultivars (**Figure 2D**). When the alkane composition of goji berries fruit was sorted for chain length groups, it was evident that the goji almost entirely participated in any monomers with chain lengths of C32, C40, and C54 (**Figure 3B**). Among them, C32 was the most abundant compound in alkane across all developmental stages. In contrast, most of the compounds with chain lengths of C40 and C54 showed a decreasing trend as the fruit matured.

#### 3.2.3 Changes in fatty acid content

Sixteen compounds were detected in goji berry fruits at four different stages of development, including 9,10,18-3-hydroxyoctadecanoic acid, 10,16-hydroxyoctadecanoic acid, and 9,10-hydroxyoctadecanoic acid (**Figure 2E**; **Supplementary Table S1**). Fatty acid was the most abundant compound in both Ningqi-1 and Ningqi-5 fruits. Ningqi-5 young fruits had the highest fatty acid content, measuring 3,012.08 ± 18.94 μg/cm^2^. As the maturity of the fruit increased, the fatty acid content in Ningqi-5 decreased to 1,259.77 ± 10.77 μg/cm^2^. Conversely, Ningqi-1 exhibited an opposite pattern: fatty acid content in Ningqi-1 young fruits was relatively low (262.79 ± 1.30 μg/cm^2^) but increased dramatically as the fruit matured, reaching 1,411.14 ± 1.87 μg/cm^2^. In fatty acid chain length analysis, it was evident that goji berry fruits almost exclusively contained monomers with chain lengths of C12–C26 (**Figure 3A**). Among them, the C24 fatty acid was particularly abundant in Ningqi-5 green fruits, with a content of 421.49 ± 28.67 μg/cm^2^, which was significantly higher than that in Ningqi-1 green fruits.

#### 3.2.4 Changes in aromatic acid and small molecule acid contents

Seven compounds were detected in goji berry fruits at four different developmental stages, mainly including 1,2,4-benzenetricarboxylic acid and *p*-coumaric acid (**Figure 2F**; **Supplementary Table S1**). The total aromatic acid and small molecule acid content in both Ningqi-1 and Ningqi-5 fruits changed substantially. As the maturity of the fruit increased, the aromatic acid and small molecule acid contents in Ningqi-1 increased from 165.97 ± 0.96 μg/cm^2^ to 1,134.14 ± 7.08 μg/cm^2^, while in Ningqi-5, they increased from 367.15 ± 13.70 μg/cm^2^ to 1,053.09 ± 16.32 μg/cm^2^. In terms of chain length, the goji berry fruits almost exclusively contained monomers with chain lengths of C4–C24 (**Figure 3C**). Notably, C12 carbon chains were only detected in Ningqi-5 young fruits. C16, C18, and C20 were the most abundant compounds in aromatic acid and small molecule acid content. The C24 content in Ningqi-5 green fruits was 421.49 ± 28.67 μg/cm^2^, which was significantly higher than that of Ningqi-1 green fruits. Focusing on aromatic acids and small molecule acids, C4 was the most abundant compound in Ningqi-5 red fruits, but it was the least abundant compound in Ningqi-1 young fruits. As the maturity of the fruit increased, aromatic acid and small molecule acid content mainly included C8, C9, and C10. Interestingly, the C24 was discovered in Ningqi-1 young fruits, but the C24 content of Ningqi-5 was the highest at 236.72 ± 19.86 μg/cm^2^.

### 3.3 Changes in cutin monomer content during goji berry development

**Figure 4:**
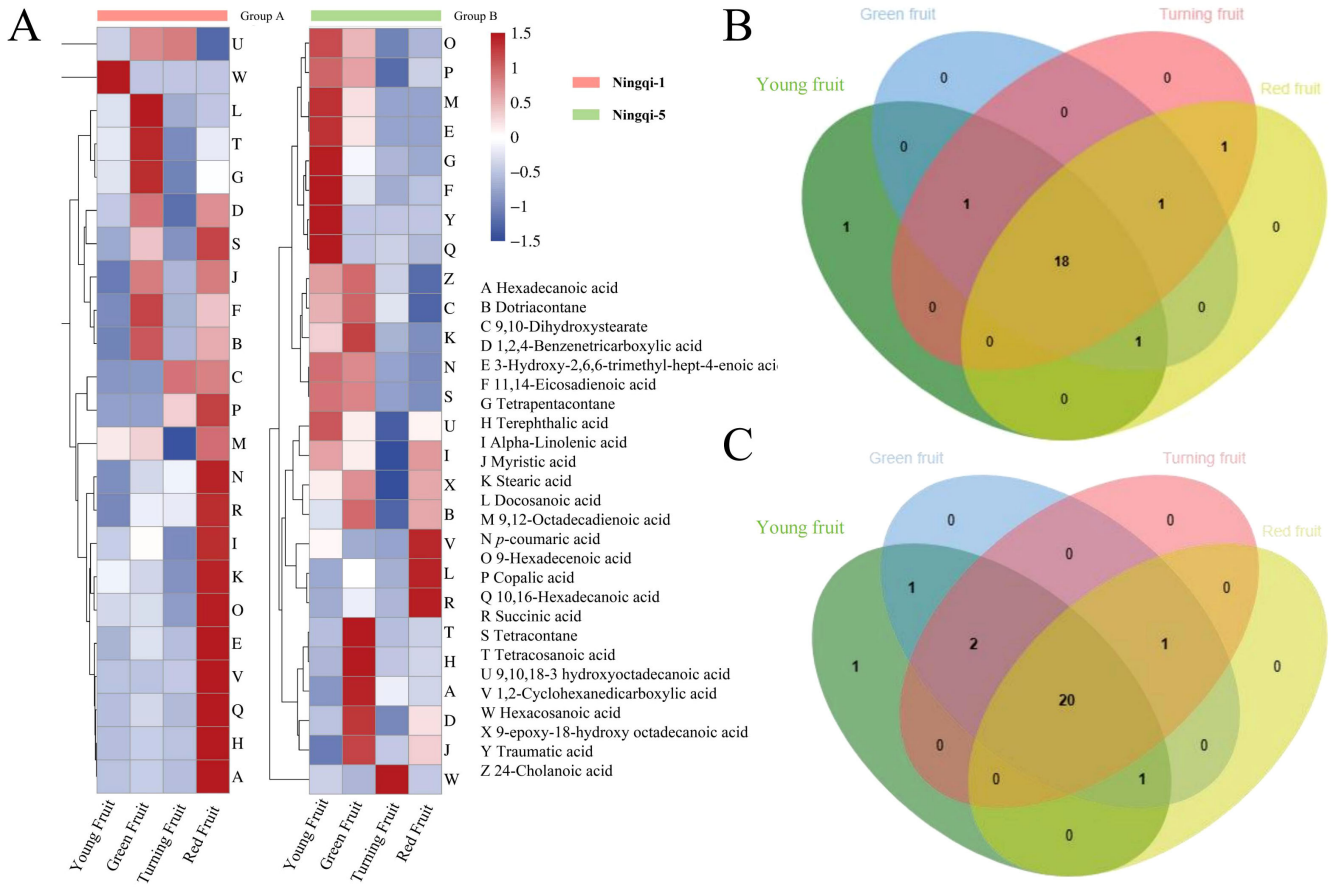
Composition and content profiles of cutin in Ningqi-1 and Ningqi-5 goji berries. **(A)** Accumulation patterns of the 26 cutin compounds. **(B, C)** Overlap of cutin compounds in Ningqi-1 and Ningqi-5 goji berries.

A total of 26 compounds were identified in Ningqi-1 and Ningqi-5 goji berries (**Supplementary Table S1**), including alkane (3), fatty acids (16), aromatic acids, and molecule acids (7). Different accumulation patterns were classified based on cluster analysis of 26 compounds in Ningqi-1 and Ningqi-5 goji berries. Hexacosanoic acid and 9,12-octadecadienoic acid exhibited the highest accumulation in young fruits, and the content of these two acids gradually decreased as the fruit matured. The highest content was found in green fruits (tetracosanoic acid, myristic acid, and dotriacontane); then, it gradually decreased (**Figure 4A**). Succinic acid and 1,2-cyclohexanedicarboxylic acid exhibited the highest content in red fruits. Eighteen compounds were conserved and collectively detected in Ningqi-1 (**Figure 4B**), and 20 compounds were conserved and collectively detected in Ningqi-5 (**Figure 4C**). Three cutin monomers were only detected in Ningqi-5.

### 3.4 Effects of goji berry cuticle extract on *A. alternata* activity *in vitro*

**Figure 5:**
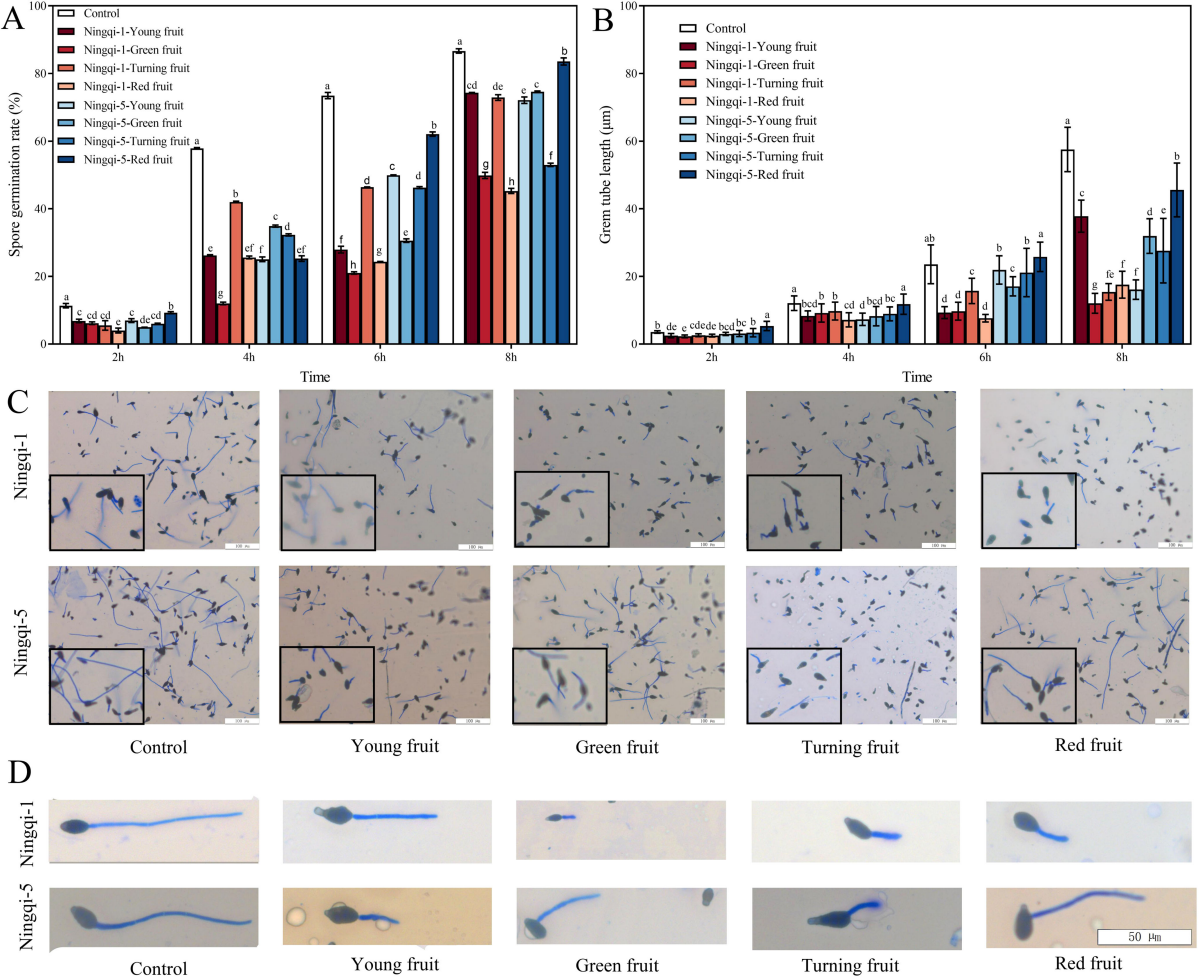
Effects of cutin extracts from goji berry fruits at different development stages on spore germination **(A, C)** and germ tube length **(B, D)**. The vertical line in the figure represents the standard error, and the lowercase letters indicate statistically significant differences between mean values during the same treatment time (*p* < 0.05).

The cuticle wax of goji berries has been shown to exhibit resistance to *A. alternata*; however, the potential effects of cutin extract on spore germination and germ tube elongation remain unclear. As shown in **Figures 5A, C**, *A. alternata* spore germination was significantly suppressed at 2 h, 4 h, 6 h, and 8 h after treatment with cutin extracts from goji berries harvested at different developmental stages, compared with control treatment. After 8 h of exposure to cutin extract from Ningqi-5 red fruits, the spore germination rate was the highest (inhibition 3.58%). In contrast, cutin extracts from Ningqi-1 red fruits exhibited the strongest inhibitory effect (inhibition 47.77%), demonstrating significant antifungal activity. The effects of cutin extracts from goji berries at different developmental stages on *A. alternata* germ tube elongation are shown in **Figures 5B, D**. Compared with the control treatment, *A. alternata* germ tube length was significantly lower after 2 h, 4 h, 6 h, and 8 h of exposure to cutin extracts. At the 8-h exposure timepoint, the cutin extract from Ningqi-5 red fruits was associated with the longest germ tube length, followed by cutin extract from Ningqi-1 young fruits, while the cutin extract from Ningqi-1 green fruits was associated with the shortest germ tube length. The length of the germ tube was inhibited of Ningqi-5 red fruits, Ningqi-1 young fruits, and Ningqi-1 green fruits by 20.79%, 69.47%, and 79.04% compared to the control treatment, respectively. In summary, the inhibitory effects of cutin extracts from goji berries on *A. alternata* germ tube elongation varied significantly at different developmental stages and cultivars. The anti-*A. alternata* effects of Ningqi-1, cutin extracts were more significant than those of Ningqi-5.

### 3.5 Effects of goji berry cutin extract on hyphal expansion of *A. alternata*

**Figure 6:**
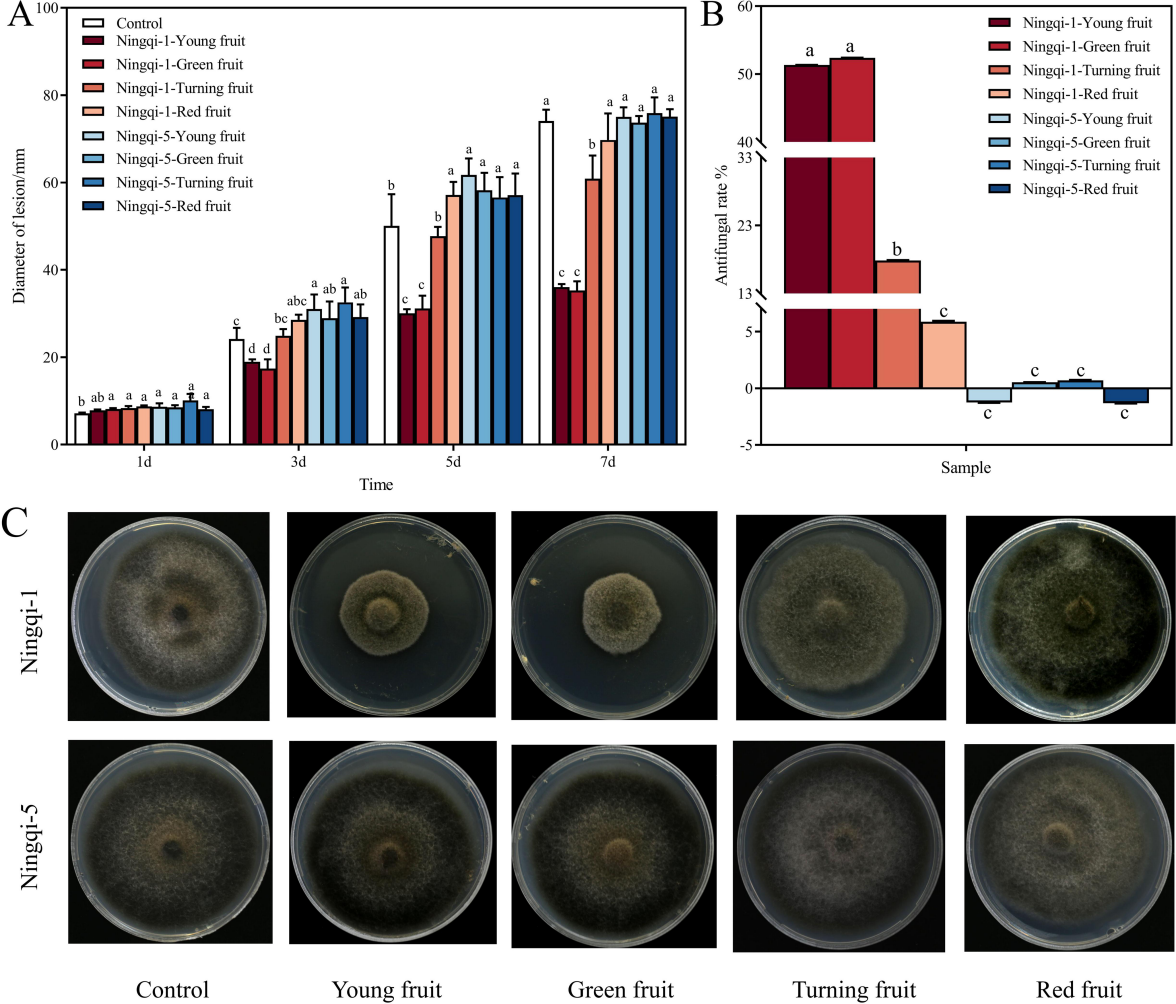
Mycelial expansion **(A)**, antifungal rates **(B)**, and phenotypic changes at 7 days **(C)** after cutin extract treatment of *Alternaria alternata* under potato dextrose agar (PDA) conditions. The vertical line in the figure represents the standard error, and the lowercase letters indicate statistically significant differences between mean values during the same treatment time (*p* < 0.05).

*In vitro* antifungal effects of goji berry cutin extracts on *A. alternata* were evaluated by measuring colony diameters using the cross-bonded method. The results revealed that the cutin extracts of Ningqi-1 young, green, and turning fruits significantly inhibited the growth of *A. alternata* compared to the control group (**Figure 6A**). Extracts from young fruits and green fruits exhibited the most significant inhibitory effects, with respective inhibition rates of 51.34% and 52.38%, respectively (**Figures 6B, C**). In contrast, the cutin extracts from Ningqi-5 had little effect on *A. alternata* mycelial expansion. The green and turning fruits had inhibitory effects on mycelial growth, and inhibitory effects of extracts from Ningqi-5 were significantly lower than those of the extracts of Ningqi-1. Extracts from goji berries at different developmental stages had different effects on *A. alternata* mycelial growth. Mycelial expansion was inhibited by cutin extract from Ningqi-1 young, green, and turning fruits. Notably, the total cutin extracts from Ningqi-1 red fruits and Ningqi-5 fruits had no effects on mycelial expansion.

### 3.6 Correlations between cutin monomer components and antifungal activity *in vitro*

**Figure 7:**
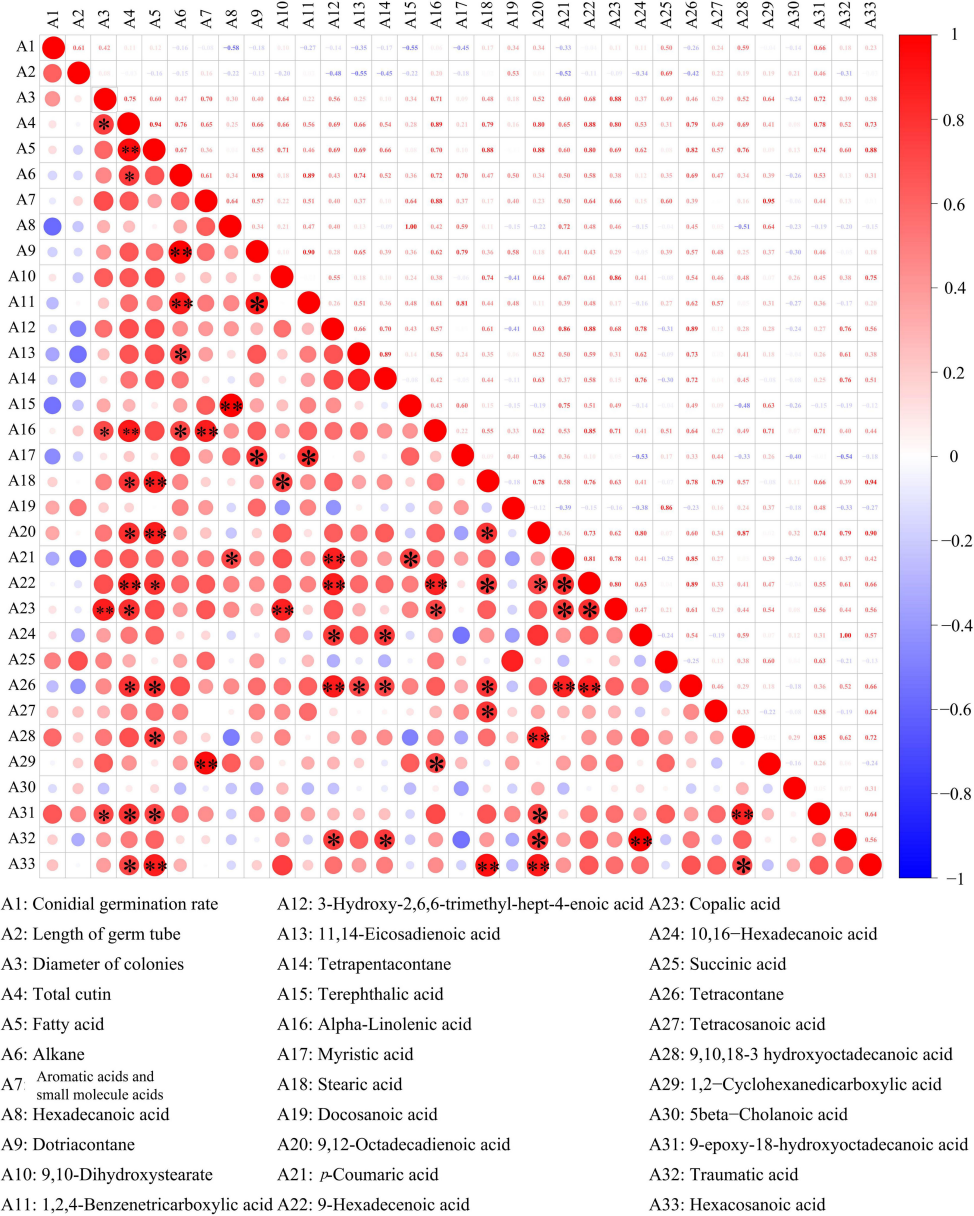
Correlations between cuticle monomer, spore germination, germ tube elongation, and mycelial expansion. *p < 0.05; **p < 0.01.

Pearson’s correlational coefficients indicated that *A. alternata* spore germination was negatively correlated with hexadecanoic acid (r^2^ = −0.58), terephthalic acid (r^2^ = −0.55), and myristic acid (r^2^ = −0.45) and positively correlated with levels of 9-epoxy-18-hydroxyoctadecanoic acid (r^2^ = 0.66), 9,10,18-3-hydroxyoctadecanoic acid (r^2^ = 0.59), and succinic acid (r^2^ = 0.50) (**Figure 7**). The germ tube length of *A. alternata* was negatively correlated with 11,14-eicosadienoic acid, tetrapentacontane, and *p*-coumaric acid and positively correlated with levels of succinic acid, docosanoic acid, and 9-epoxy-18-hydroxy octadecanoic acid. Most of the substances in the cutin monomer components of goji berry were positively correlated with *A. alternata* mycelial expansion. Among them, 9-epoxy-18-hydroxy octadecanoic acid and copalic acid had significant effects. However, there were no significant correlations between total cutin content, total alkanes, total fatty acids, total aromatic acids and small molecule acids, and spore germination and germ tube length.

### 3.7 *In vitro* antifungal activity of different goji berry monomeric cutin fractions

**Figure 8:**
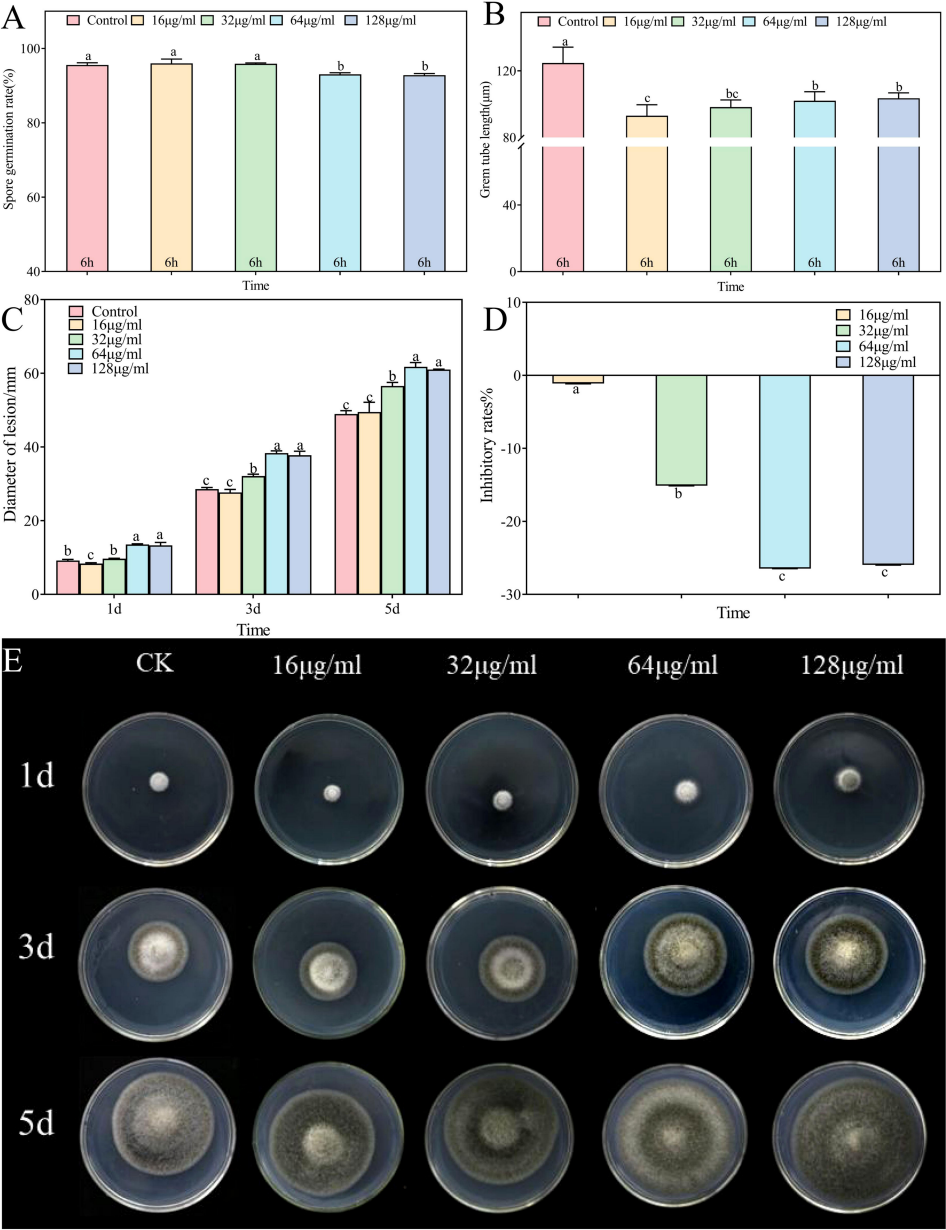
*Alternaria alternata* was affected by palmitic acid. **(A)** Spore germination. **(B)** Germ tube length. **(C)** Mycelial expansion. **(D)** Inhibition rate. **(E)** Phenotypic changes. The vertical line in the figure represents the standard error, and the lowercase letters indicate statistically significant differences between mean values during the same treatment time (*p* < 0.05).

**Figure 9:**
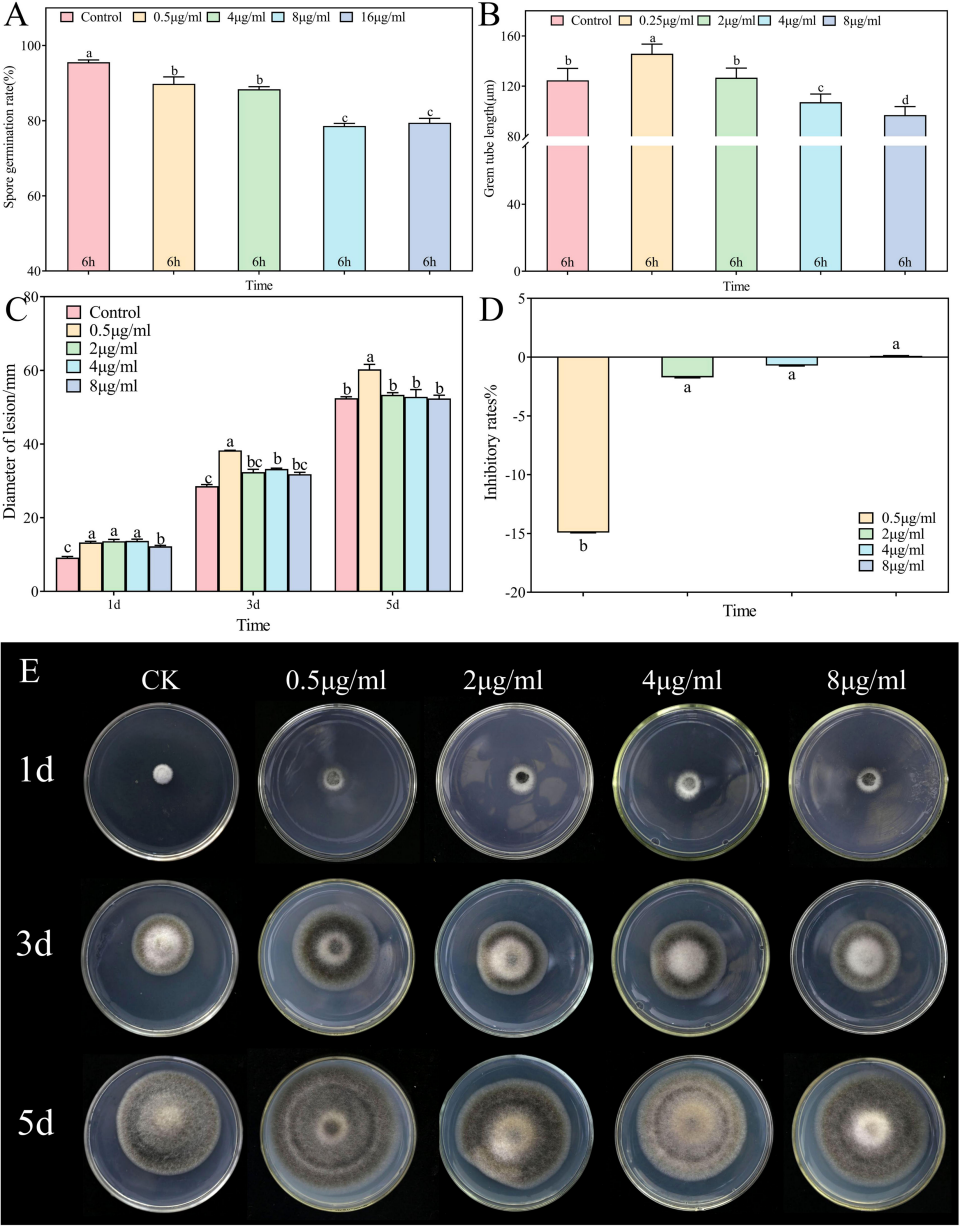
*Alternaria alternata* was affected by linoleic acid. **(A)** Spore germination. **(B)** Germ tube length. **(C)** Mycelial expansion. **(D)** Inhibition rate. **(E)** Phenotypic changes. The vertical line in the figure represents the standard error, and the lowercase letters indicate statistically significant differences between mean values during the same treatment time (*p* < 0.05).

**Figure 10:**
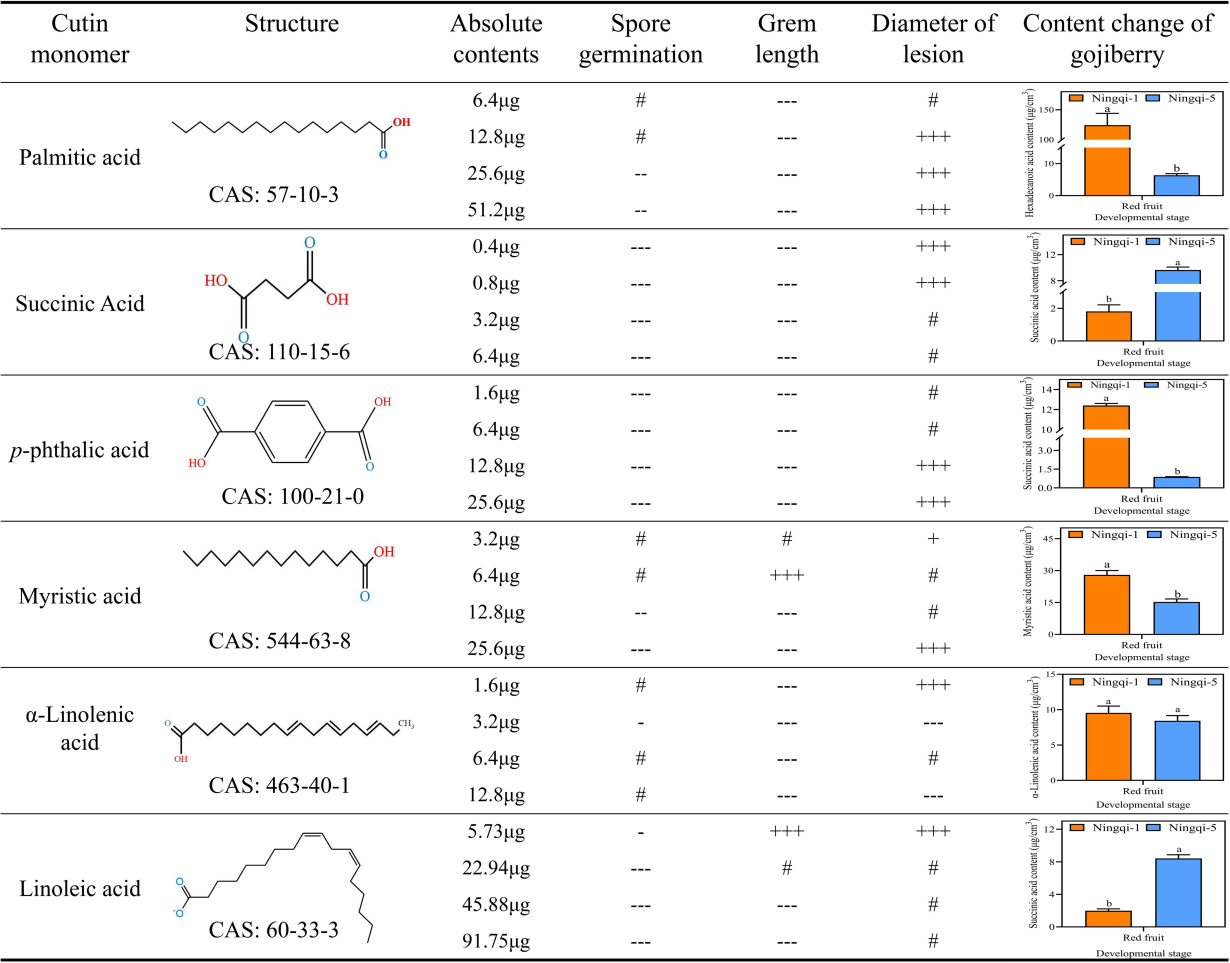
Correlations between cuticle monomer, spore germination, germ tube elongation, and mycelial expansion. Compared with the control condition: “+” *p*< 0.05; “++” *p* ≤ 0.01; “+++” *p* ≤ 0.001; “-” *p*< 0.05; “--” *p*≤ 0.01; “---” *p* ≤ 0.001; “#” not significant. + represent positive correlation, - represent negative correlation.

*In vitro* assessments of the anti-*A*. alternata activity of palmitic acid, myristic acid, linoleic acid, α-linolenic acid, succinic acid, and terephthalic acid revealed that the effects of palmitic acid and linoleic acid were the most significant (**Figures 8**, **9**, **10**; **Supplementary Figures S1**-**S4**). The minimum inhibitory concentration (MIC) of linoleic acid was 8.21 μg/mL. *A. alternata* spore germination was not significantly affected by various treatments with palmitic acid (**Figure 8A**). However, germ tube elongation was significantly inhibited. Germ tube elongation reached 124.67 ± 9.51 μm in the control condition after incubation for 6 h, but treatment with 32 μg/mL and 64 μg/mL of palmitic acid inhibited germ tube elongation, with inhibition rates of 18.16% and 21.18%, respectively (**Figure 8B**). Mycelial growth of *A. alternata* in the PDA medium was significantly promoted by palmitic acid in a centration-dependent manner (**Figure 8C**). For example, in the presence of 64 μg/mL palmitic acid, mycelial growth was 20.93% higher than that of the control (**Figures 8D, E**). *A. alternata* spore germination reached 95.56% under the control condition after 6-h incubation. Both spore germination and germ tube length on the PDA medium were significantly promoted by linoleic acid in a centration-dependent manner (**Figures 9A, B**). Compared to the control, mycelial growth in the presence of 0.5 μL/mL linoleic acid was significantly enhanced. However, higher concentrations of linoleic acid (2 μL/mL, 4 μL/mL, and 8 μL/mL) had no significant effect on mycelial growth compared to the control (**Figure 9C**). After 5 days of incubation, mycelial growth with 8 μg/mL linoleic acid was slightly reduced by 0.11% compared to the control (**Figures 9D, E**).

## 4 Discussion

Cuticular wax is a complex mixture that covers the plant cuticle and plays an important role in the biosynthesis of cutin building blocks (Girard et al., 2012; Segado et al., 2020; Aloui et al., 2021). Recent studies have described the structure and thickness of cuticular wax in tea leaves, highlighting its potential positive function with reference to antifungal properties and natural resources in bionics (Altieri et al., 2007; Ding et al., 2018; Zhu et al., 2018). However, the content and composition of cutin, which accumulates in goji berries at different developmental stages, and their role in antifungal properties remain unclear. In our study, fluctuations in cutin content, composition, and cuticular microstructure in goji berries were monitored at different growth stages. Additionally, the relationship between specialized cutin monomer compounds and antifungal capacity was systematically investigated.

Many researchers have reported differences in cutin chemical composition and structure in different tissues that result in differences in the basic characteristics of cuticles in those tissues (Järvinen et al., 2010; Fich et al., 2016; Wang et al., 2016; Fawke et al., 2019; Wu et al., 2022). In a previous study, when chloroform was used to remove cuticular wax from a plant, optical microscopy and transmission electron microscope (TEM) revealed a dense structure, which was suggested to be rich in cutin monomers (Shumborski et al., 2016). Liu et al. (2024) monitored changes in the cell structure of peels from different pumpkin varieties using paraffin sections and found that the cuticle was uniformly and continuously distributed outside the cell wall and gradually thinned during the late developmental stage. Based on TEM, structurally, the plant cuticle has been described as having two distinct zones: one comprised of monomeric waxes and the other of cutin polyester. In this study, the cutin structure in developing goji berry fruits was examined in paraffin sections. Ningqi-1 cells displayed higher density and stronger adhesion to adjacent cells, which is consistent with findings from a previous study on apples (Ginzberg and Stern, 2019). It has also been reported that fruit cracking was associated with the spatio-temporal development of epidermal cell density and cutin thickness (Demirsoy and Demirsoy, 2004; Onoda et al., 2012). Our results showed that differences in cuticle thickness were observed at the different developmental stages of goji berries. We speculate that Ningqi-1 red fruits are less likely to crack than Ningqi-5 red fruits. However, compared with the other three developmental stages, red fruits were less likely to be preserved.

Differences in cutin structure can be detected via microscopy, and its chemical components of cutin can be analyzed via GC/MS. Cutin is a polyester structure mainly composed of C16 and C18 fatty acid derivatives, characterized by one or more hydroxyl groups, medium-chain ring oxides, and terminal carboxyl functional groups (Pollard et al., 2008). Previous studies have shown that the chemical composition and content of cutin vary with the spatio-temporal development of fruit and the growth environment, and the total cutin content can make a large difference in each species (Xu et al., 2014; Huang et al., 2017). In *Olea europaea* L., ω-hydroxy fatty acids predominated in fruit cutin (Huang et al., 2017). The ‘Somerset’ and ‘Celeste’ cherry fruit varieties were mainly composed of C18 monomers (65.3%) (Belge et al., 2014). Kosma et al. (2010) showed an increase in total cutin monomers for tomato mutants during the fruit development stage, which was consistent in Ningqi-1 fruit. Generally, there are significant differences in cutin content in different fruits. It is reasonable that there are differences in the total cutin content obtained from the different characteristics of the fruit, as the methods of sampling and surface area calculation are different (Wu et al., 2018). In addition, the cutin thickness and content in Ningqi-5 were higher than those in Ningqi-1, which was consistent with previous studies on pepper fruit. It was shown that *Vezena Slatka* fruits exhibited modified skin anatomy and structure and significantly higher cutin content compared to Numex Garnet fruits from 20 days after anthesis (daa) until full maturity at 60 daa (Marinov et al., 2023). Qin et al. (Zhang et al., 2021) and Zhi et al. (2024)) showed that the difference in cutin composition between Ningqi-1 and Ningqi-5 may be due to the high-efficiency expression of several key genes (Zhang et al., 2021; Zhi et al., 2024). Furthermore, goji berries exhibited both high fatty acid content and high alkane content, which was consistent to cherry tomato, *Malus domestica*, and *Prunus avium* fruit studied (Matas et al., 2004; Lai et al., 2016). This confirms the conservation of cutin components to an extent.

Previous studies have demonstrated that cuticle function is affected by compositional and proportional changes in cutin monomer content (Marga et al., 2001; Kosma et al., 2010; Ding et al., 2020). Marga et al. (2001) showed that the cutin of rigid tissues can be classified as the Cl6 type, whereas the cutin of elastic tissue corresponds to a mixed C16/C18 type. Kosma et al. (2010) and Marga et al. (2001) discovered that the cutin of elastic tissues has a higher trihydroxy fatty acid content. Here, we demonstrated that Ningqi-1 exhibited a higher C16/C18 ratio compared to Ningqi-5 during the same developmental stage, which was not consistent with previous studies in tomato. It showed that no biomechanical differences were detected in the cuticles of young fruit, while reduced elasticity became evident at the mature stage (Bargel and Neinhuis, 2005; López-Casado et al., 2007; Domínguez et al., 2009). Knoche et al. (2004) found that the onset of elastic strain coincided with the cessation of cuticular membrane information of sweet cherry (*P. avium* L.) fruit. However, the physical and chemical mechanisms by which the ratio of C16/C18 and hydroxylated cuticle monomers regulate the mechanical properties of cutin remain unclear (Marga et al., 2001; Graca et al., 2002; Khanal and Knoche, 2017).

Pathogen resistance during fruit development is influenced by factors such as fungal enzymes (pectinase and cutinase), cuticle structure and thickness, antifungal compound levels, nutrient availability, and resistance gene expression at various growth stages (Mahasuk et al., 2009; Mahasuk et al., 2013). The sensitivity of goji berries to *A. alternata* is influenced by the composition and content of cuticle wax (Wang et al., 2021). However, the role of cutin in resisting *A. alternata* infestation remains unclear. Our results demonstrated that spore germination rate and germ tube elongation of *A. alternata* were significantly inhibited by goji berry cutin extracts, confirming previous findings that cutin plays a vital role in protecting plant tissues from microbial infection (Isaacson et al., 2009). Another study revealed that Lbg/cutin monomers reduced the fungal infection rate of tomatoes in the range of 55%–60% at 4 dpi (Aloui et al., 2021). Notably, fatty acids, alkanes, aromatic acids, and small molecule acids were the major components of goji berry cutin. The cutin composition of goji berries contains a variety of substances, such as *p*-coumaric acid and tetradecanoic acid, which are known for their high antifungal potential in inhibiting the growth of *Botrytis cinerea* (Vio-Michaelis et al., 2012; Hapon et al., 2017). In addition, there are several reports that *p*-coumaric acid can inhibit *A. alternata* growth (Li et al., 2018; Yuan et al., 2019). Interestingly, *p*-coumaric acid and myristic acid accumulated rapidly during the gradual maturity of Ningqi-1 fruit, as opposed to Ningqi-5. These findings indicated that myristic acid and *p*-coumaric acid composition of cutin accumulation and composition regulate the resistance of goji berries.

In recent years, the bactericidal and antifungal activities of fruit cutin oligomers and monomers toward *Escherichia coli*, *Staphylococcus aureus*, and *Venturia inaequalis* have been discovered (Escórcio et al., 2022; Gaucher et al., 2024). Several studies have shown that fatty acids have antifungal activity, and the antifungal ability was closely related to the structural characteristics and targeted pathogens of fatty acids (Pohl et al., 2011; Guimarães and Venâncio, 2022; Gaucher et al., 2024). During the fruit growth of goji berries, fatty acids increased in Ningqi-5 and decreased in Ningqi-1 yet remained the most abundant substances overall. It has been demonstrated that high concentrations of palmitic acid and linoleic acid significantly inhibited fungal spore germination and germ tube elongation, consistent with our *in vitro* experiments (Altieri et al., 2007; Chu et al., 2014). It has long been known that cutin monomers have antifungal activity (Walters et al., 2004; Wu et al., 2011; Li et al., 2018; Yuan et al., 2019). Correlation analysis further demonstrated that the cutin monomers of goji berries are associated with the growth and development of *A. alternata*. However, no evidence was found that the accumulation and composition of cutin directly regulate the resistance of goji berries, warranting further investigation.

**Figure 11:**
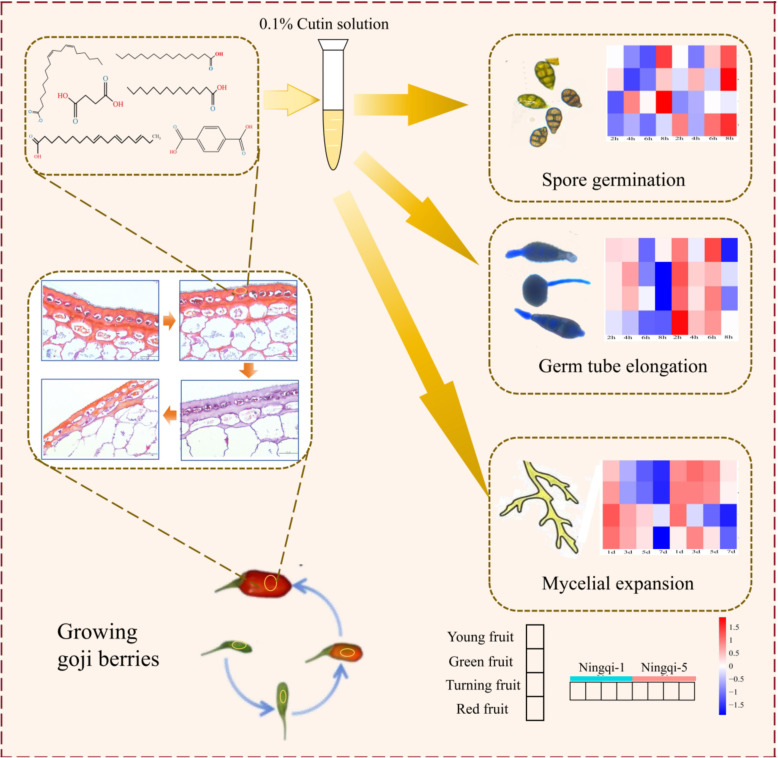
Summary of effects of total goji berry cutin extract on *Alternaria alternata* spore germination, germ tube elongation, and mycelial expansion.

In summary, our study investigated the compositions, characteristics, and antifungal capacities of cutin in goji berry fruits at different stages of development. Ningqi-1 exhibited a dense cuticle structure and high cell density. GC/MS analysis revealed that fatty acids, alkanes, aromatic acids, and small molecule acids are the major components of goji berry cutin. Cutin extracts inhibited the growth of *A. alternata*. Among the identified substances, α-linolenic acid, hexadecanoic acid, succinic acid, and *p*-phthalic acid can be used as potential antifungal compounds present in goji berries (**Figure 11**). These findings highlight the potential role of cutin in enhancing resistance to *A. alternata* and provide a basis for further research on its functional characteristics and accumulation mechanisms during goji berry development.

## Data Availability

The original contributions presented in the study are included in the article/**Supplementary Material**. Further inquiries can be directed to the corresponding authors.

## Supplementary material

Supplementary Figure 1
*A.alternata* was affected by p-phthalic acid. **(A)** Spore germination. **(B)** Germ tube length. **(C)** Mycelial expansion. **(D)** Inhibitory rate. **(E)** Phenotypic changes. The vertical line in the figure represents the standard error, and the lowercase letters indicate statistically significant differences between mean values during the same treatment time (*p* < 0.05).

Supplementary Figure 2
*A.alternata* was affected by succinic acid. **(A)** Spore germination. **(B)** Germ tube length. **(C)** Mycelial expansion. **(D)** Inhibitory rate. **(E)** Phenotypic changes. The vertical line in the figure represents the standard error, and the lowercase letters indicate statistically significant differences between mean values during the same treatment time (*p* < 0.05).

Supplementary Figure 3
*A.alternata* was affected by myristic acid. **(A)** Spore germination. **(B)** Germ tube length. **(C)** Mycelial expansion. **(D)** Inhibitory rate. **(E)** Phenotypic changes. The vertical line in the figure represents the standard error, and the lowercase letters indicate statistically significant differences between mean values during the same treatment time (*p* < 0.05).

Supplementary Figure 4
*A.alternata* was affected by α-linolenic acid. **(A)** Spore germination. **(B)** Germ tube length. **(C)** Mycelial expansion. **(D)** Inhibitory rate. **(E)** Phenotypic changes. The vertical line in the figure represents the standard error, and the lowercase letters indicate statistically significant differences between mean values during the same treatment time (*p* < 0.05).
