# Supplementary figures and images for: Analysis of the composition, characteristics, and antifungal properties of cutin in goji berry fruits at different developmental stages
Source: Front Plant Sci. 2025 Feb 11;16:1528881. doi: 10.3389/fpls.2025.1528881 (PMC11850552; doi:10.3389/fpls.2025.1528881)

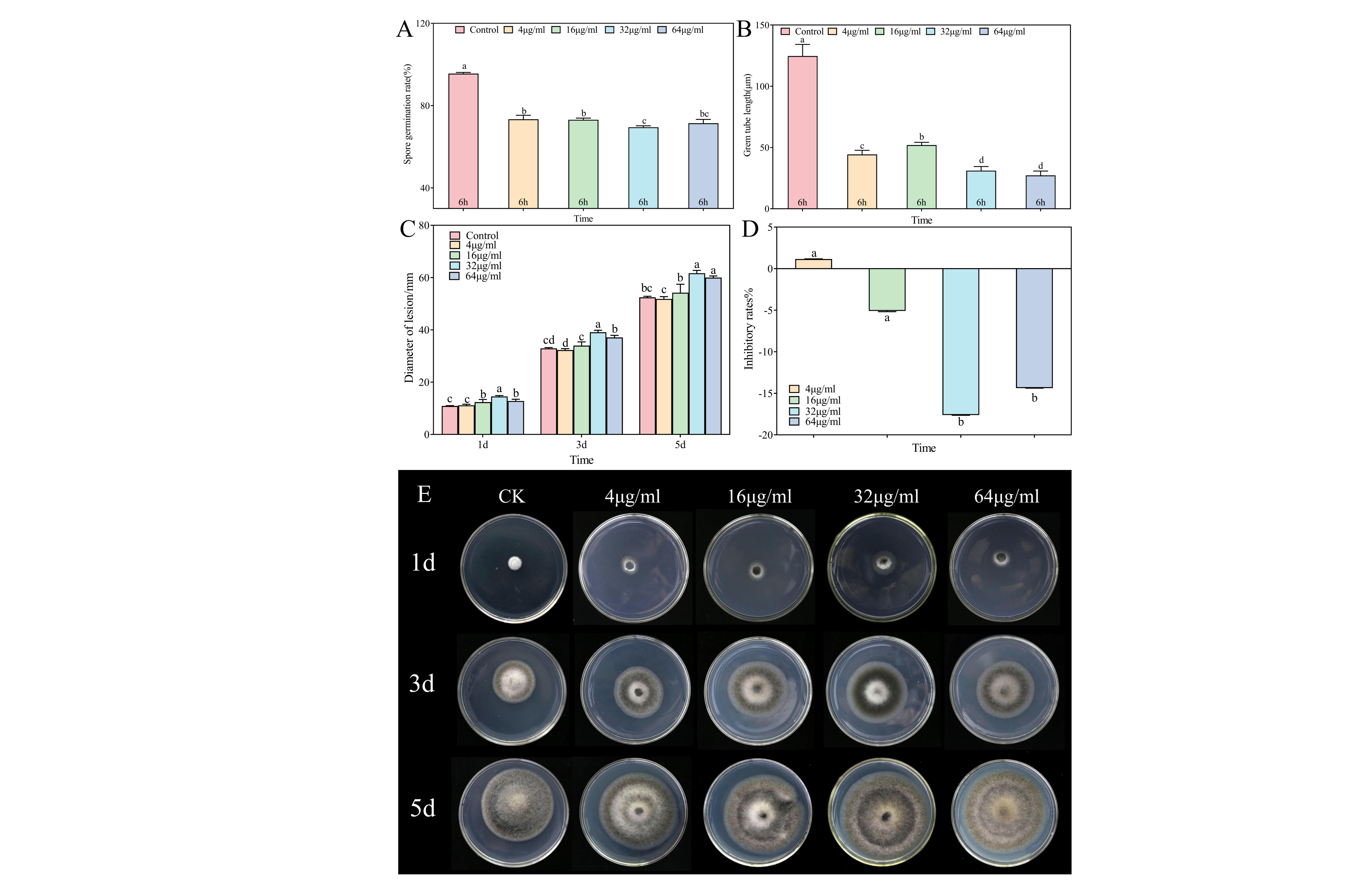

Supplement: Supplementary Figure 1 — A.alternata was affected by p-phthalic acid. (A) Spore germination. (B) Germ tube length. (C) Mycelial expansion. (D) Inhibitory rate. (E) Phenotypic changes. The vertical line in the figure represents the standard error, and the lowercase letters indicate statistically significant differences between mean values during the same treatment time (p < 0.05). [file Image1.jpeg]

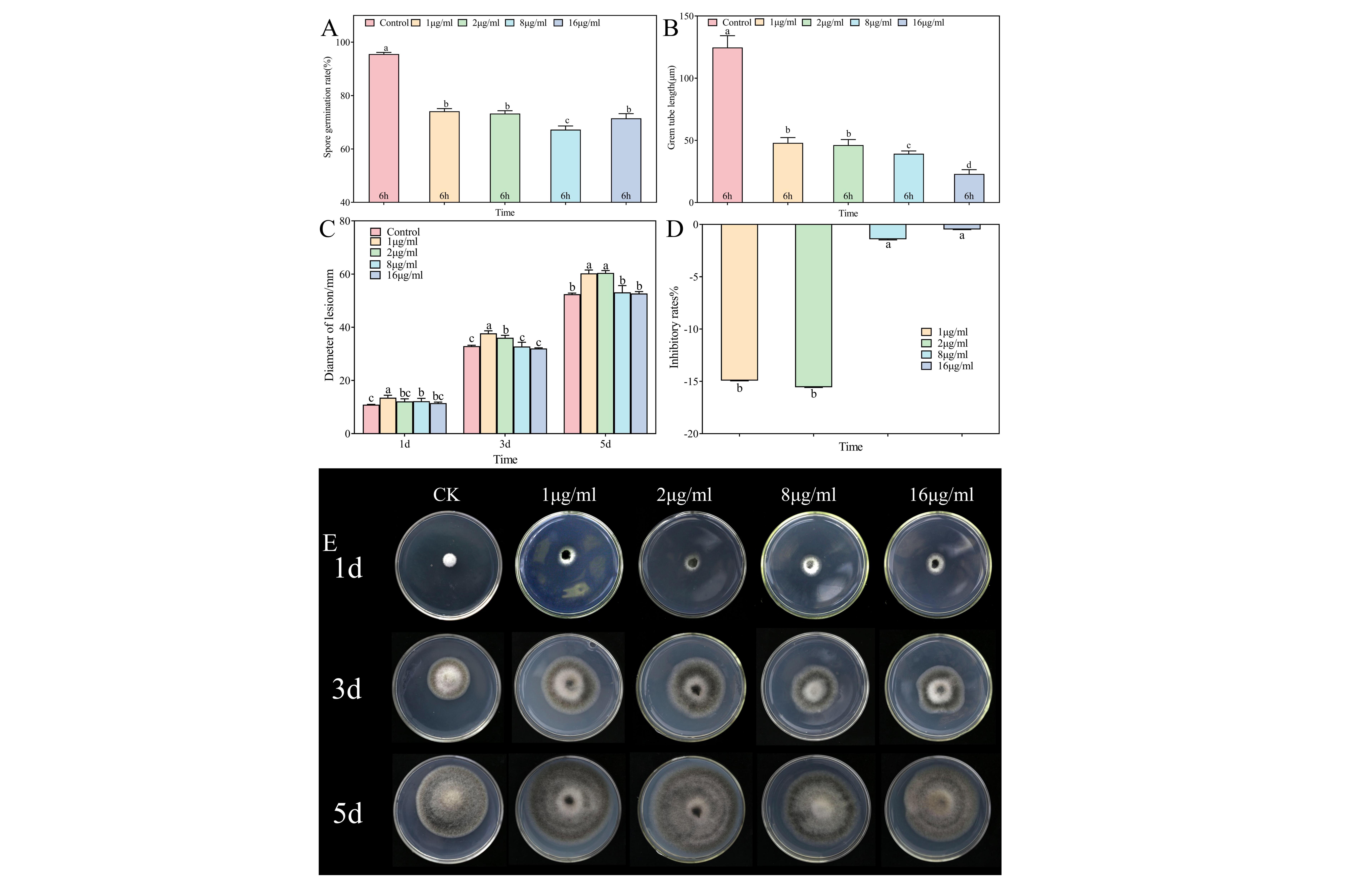

Supplement: Supplementary Figure 2 — A.alternata was affected by succinic acid. (A) Spore germination. (B) Germ tube length. (C) Mycelial expansion. (D) Inhibitory rate. (E) Phenotypic changes. The vertical line in the figure represents the standard error, and the lowercase letters indicate statistically significant differences between mean values during the same treatment time (p < 0.05). [file Image2.jpeg]

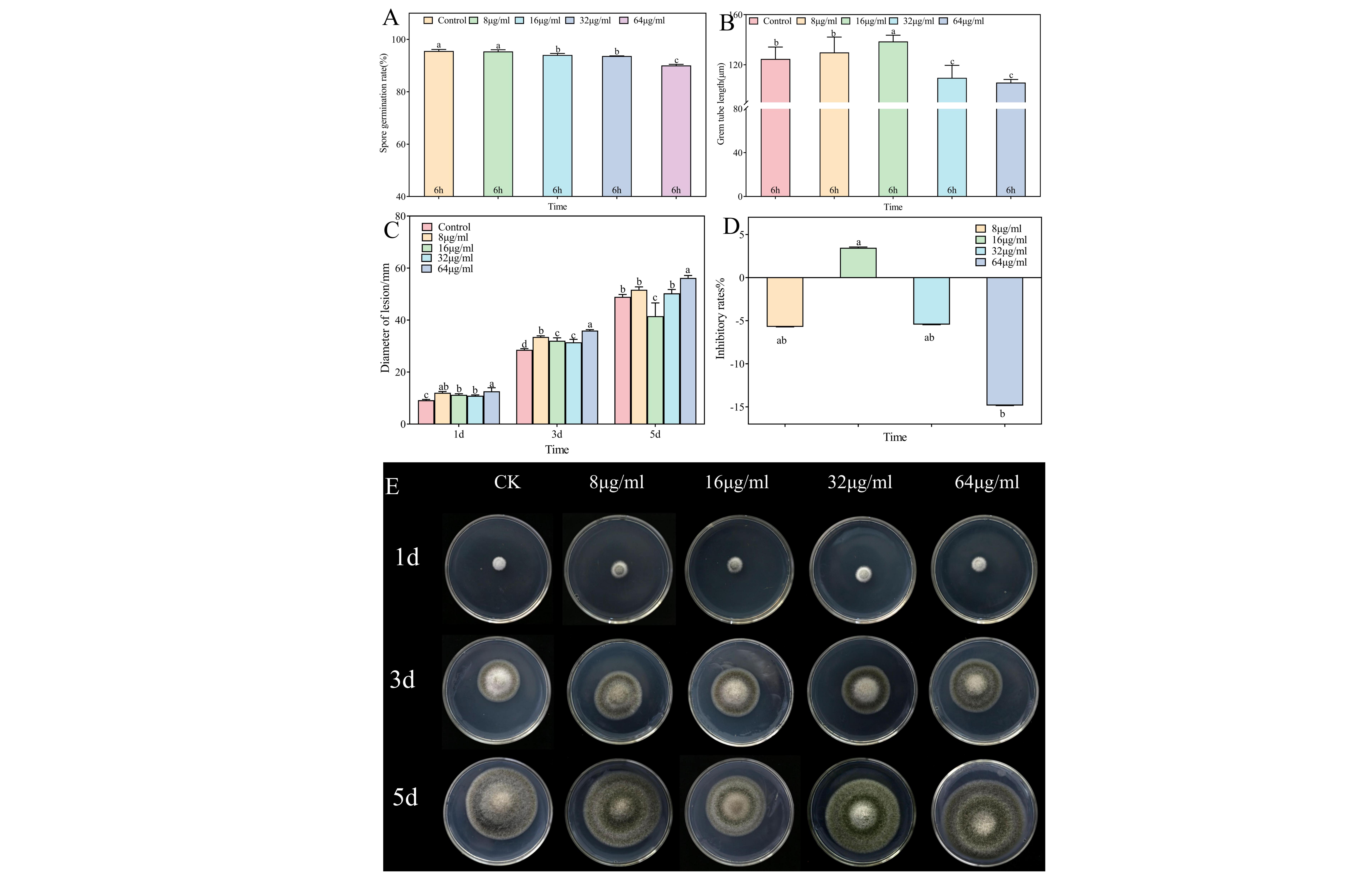

Supplement: Supplementary Figure 3 — A.alternata was affected by myristic acid. (A) Spore germination. (B) Germ tube length. (C) Mycelial expansion. (D) Inhibitory rate. (E) Phenotypic changes. The vertical line in the figure represents the standard error, and the lowercase letters indicate statistically significant differences between mean values during the same treatment time (p < 0.05). [file Image3.jpeg]

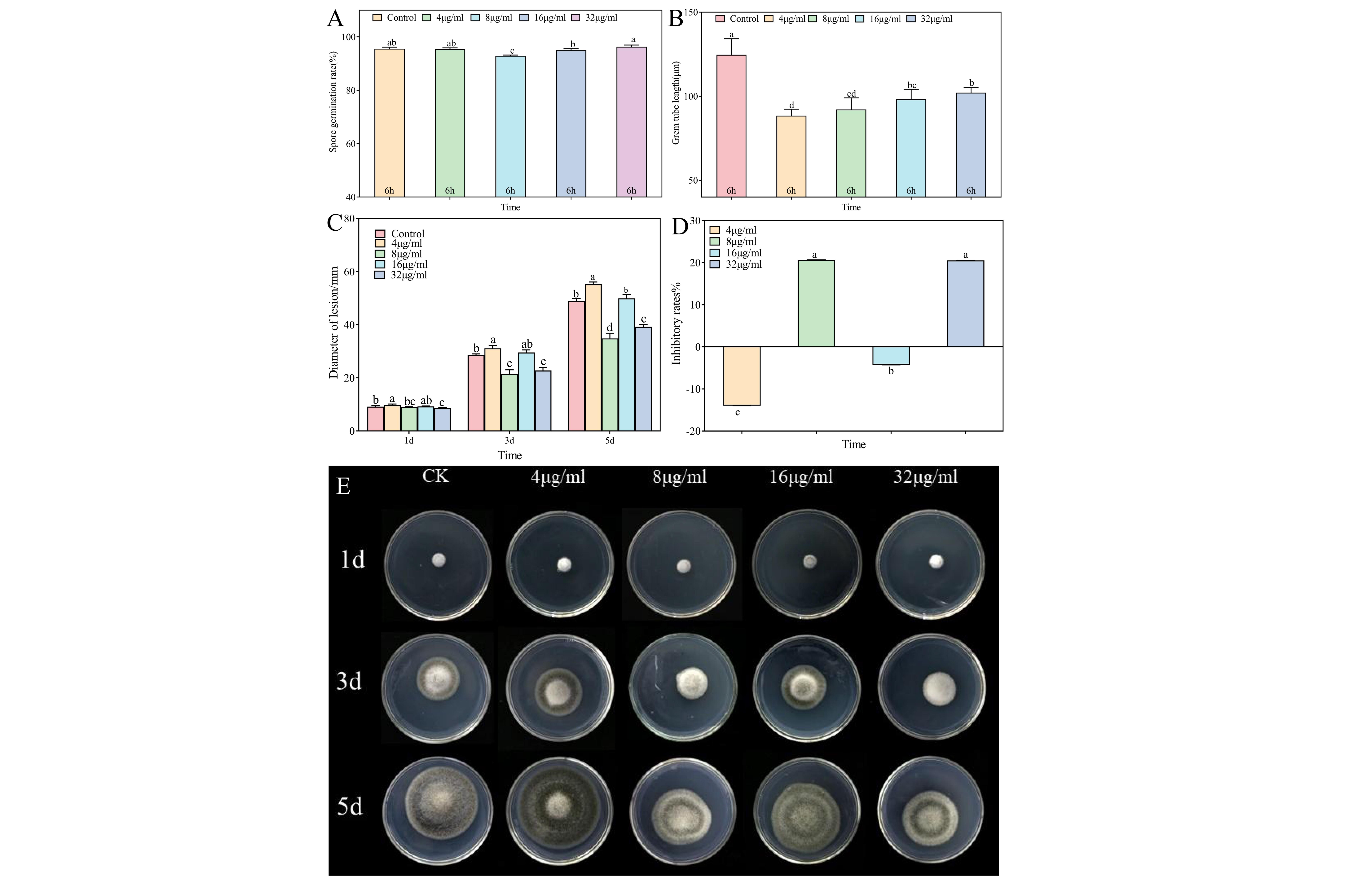

Supplement: Supplementary Figure 4 — A.alternata was affected by α-linolenic acid. (A) Spore germination. (B) Germ tube length. (C) Mycelial expansion. (D) Inhibitory rate. (E) Phenotypic changes. The vertical line in the figure represents the standard error, and the lowercase letters indicate statistically significant differences between mean values during the same treatment time (p < 0.05). [file Image4.jpeg]
