# Supplementary material for: Analysis of the composition, characteristics, and antifungal properties of cutin in goji berry fruits at different developmental stages
Source: Front Plant Sci. 2025 Feb 11;16:1528881. doi: 10.3389/fpls.2025.1528881 (PMC11850552; doi:10.3389/fpls.2025.1528881)
Supplement: Supplementary file 5 [file Table1.docx]

Supplementary Table 1. Chemical composition of the cutin from the different developmental stages of two *Lycium barbarum L* cultivars.

| Compounds | RI | RI* | Ningqi-1 | | | | Ningqi-5 | | | |
| --- | --- | --- | --- | --- | --- | --- | --- | --- | --- | --- |
|  |  |  | Young fruit | Green fruit | Turning fruit | Red fruit | Young fruit | Green fruit | Turning fruit | Red fruit |
| Hexadecanoic acid | 2382.00 | 2712.43 | 19.65±0.65 | 62.97±3.69 | 11.05±1.40 | 839.98±133.15 | 15.00±1.51 | 187.41±19.14 | 67.02±12.50 | 51.51±0.98 |
| Dotriacontane | 3203.00 | 3031.20 | 170.92±20.06 | 670.08±125.17 | 268.93±85.33 | 546.09±51.95 | 417.20±9.43 | 626.59±43.19 | 249.76±16.87 | 559.61±22.23 |
| 9,10-Dihydroxystearate | 2784.00 | 3050.53 | 26.35±2.61 | 37.08±1.49 | 485.91±85.97 | 458.46±91.66 | 612.20±90.67 | 711.52±87.59 | 445.70±14.53 | 229.43±28.83 |
| 1,2,4-Benzenetricarboxylic acid | 1238.00 | 1262.34 | 86.83±5.95 | 226.80±37.08 | 12.49±5.14 | 208.44±3.24 | 105.17±2.48 | 242.21±13.35 | 70.47±4.92 | 161.62±10.99 |
| 11,14-Eicosadienoic acid | 2401.00 | 2661.84 | Nd | 154.98±31.52 | 23.13±14.27 | 97.05±19.27 | 169.80±24.70 | 79.81±2.30 | 55.75±7.72 | 64.57±9.08 |
| Tetrapentacontane | 5400.00 | 5858.34 | 27.71±2.48 | 77.90±6.5 | 3.69±0.90 | 33.99±0.27 | 106.18±43.48 | 44.52±6.29 | 23.97±1.68 | 18.70±1.47 |
| Terephthalic acid | 1658.00 | 2027.62 | 3.97±0.18 | 8.26±1.94 | 5.03±0.80 | 83.86±1.34 | 5.18±0.35 | 23.76±5.58 | 6.06±0.08 | 7.09±0.14 |
| α-Linolenic acid | 2210.00 | 2787.69 | 19.63±1.39 | 32.11±1.76 | 6.80±0.29 | 64.49±6.56 | 67.09±8.34 | 59.00±7.22 | 32.49±4.14 | 68.05±6.01 |
| Myristic acid | 1788.00 | 2133.49 | 4.99±0.89 | 27.97±3.77 | 10.76±4.56 | 27.98±2.02 | Nd | 24.60±2.75 | 6.65±0.29 | 15.26±1.40 |
| Stearic acid | 2186.00 | 2379.49 | 7.36±0.30 | 6.36±1.08 | 3.84±0.91 | 14.60±0.01 | 29.46±3.37 | 44.70±3.82 | 12.15±1.31 | 6.75±1.00 |
| Docosanoic acid | 2475.00 | 2775.08 | 16.00±0.04 | 30.62±0.50 | 12.29±2.26 | 14.15±3.34 | 9.95±3.91 | 23.04±9.38 | 10.82±1.01 | 48.03±5.91 |
| 9,12-Octadecadienoic acid | 2292.00 | 2427.24 | 8.99±1.31 | 9.77±0.56 | Nd | 13.46±1.54 | 217.13±23.85 | 139.31±8.16 | 68.06±10.56 | 67.95±3.72 |
| *p*-Coumaric acid | 1565.00 | 2006.93 | 22.10±2.65 | 35.81±6.10 | 41.40±8.66 | 77.92±17.89 | 62.43±2.31 | 59.00±0.71 | 28.89±2.41 | 24.88±1.79 |
| 9-Hexadecenoic acid | 2209.00 | 2493.95 | 13.81±0.67 | 14.14±1.07 | 5.80±2.36 | 43.28±10.64 | 50.69±4.73 | 40.40±1.25 | 15.60±1.22 | 22.60±4.18 |
| Copalic acid | 3733.00 | 3733.62 | Nd | Nd | 92.57±5.50 | 164.59±41.76 | 168.66±27.46 | 152.28±4.66 | 75.61±13.07 | 109.03±10.76 |
| 10,16-Hexadecanoic acid | 2382.00 | 2984.09 | 13.90±2.87 | 19.60±5.76 | 12.67±1.70 | 67.78±14.52 | 828.58±124.91 | 33.06±7.45 | 77.90±7.99 | Nd |
| Succinic acid | 1963.00 | 1963.35 | 2.47±0.59 | 5.96±2.11 | 5.82±2.11 | 12.24±2.71 | 4.29±0.29 | 22.40±0.68 | 7.14±1.10 | 77.93±3.58 |
| Tetracontane | 3997.00 | 4164.78 | 10.21±1.68 | 21.82±1.34 | 8.30±0.78 | 30.02±5.27 | 33.96±4.04 | 32.51±3.52 | 11.04±1.82 | 8.91±0.91 |
| Tetracosanoic acid | 2674.00 | 3064.26 | 38.20±6.93 | 70.66±0.47 | 23.82±6.41 | 38.99±8.57 | 20.19±0.06 | 490.28±40.12 | 16.28±2.25 | 55.39±0.57 |
| 9,10,18-3hydroxyoctadecanoic acid | 2801.00 | 3009.88 | 118.34±21.30 | 281.54±70.27 | 297.31±6.71 | Nd | 827.99±23.01 | 642.67±3.90 | 365.94±12.61 | 635.69±37.04 |
| 1,2-Cyclohexanedicarboxylic acid | 3070.00 | 3105.90 | 10.39±0.88 | 12.71±0.75 | 34.09±3.29 | 794.13±143.05 | 290.63±83.94 | 41.73±6.42 | 25.55±4.49 | 719.90±21.42 |
| 3-Hydroxy-2,6,6-trimethyl-hept-4-enoic acid | 1393.00 | 2938.34 | 23.11±2.68 | 43.22±9.59 | 29.29±12.14 | 142.71±32.12 | 207.50±30.07 | 94.97±19.18 | Nd | Nd |
| 24-Cholanoic acid | 2790.00 | 2943.26 | 4.76±1.00 | Nd | Nd | Nd | 64.69±9.63 | 47.25±5.89 | 236.72±19.86 | 59.37±10.27 |
| 9-epoxy-18-hydroxy octadecanoic acid | 2413.00 | 2937.60 | Nd | Nd | Nd | Nd | 90.36±4.72 | 123.63±5.93 | Nd | 115.63±23.85 |
| Traumatic acid | 1973.00 | 3161.03 | Nd | Nd | Nd | Nd | 309.45±26.73 | Nd | Nd | Nd |
| Hexacosanoic acid | 2872.00 | 3496.76 | Nd | Nd | Nd | Nd | 10.73±1.05 | 12.49±0.81 | 4.55±1.05 | Nd |

Note: The data in Supplementary Table 1. represents the cutin content (μg/cm^2^) of goji berries, all of which are average ± standard deviation (SD). RI*is the retrieved value of NIST17, and RI** is the measured value. Nd, not detected.
